# Association of Serum Adropin Concentrations with Diabetic Nephropathy

**DOI:** 10.1155/2016/6038261

**Published:** 2016-07-28

**Authors:** Wenchao Hu, Li Chen

**Affiliations:** ^1^Department of Endocrinology, Qilu Hospital, Shandong University, Qingdao, China; ^2^Department of Endocrinology, Qilu Hospital, Shandong University, 107 West Wenhua Road, Jinan, Shandong 250012, China

## Abstract

*Objective*. Adropin is a newly identified regulatory protein encoded by the Enho gene and is critically involved in energy homeostasis and insulin sensitivity. This study aims to determine the correlation of serum adropin concentrations with diabetic nephropathy (DN).* Methods*. This study consisted of 245 patients with type 2 diabetes mellitus (T2DM) and 81 healthy subjects. Then T2DM patients were divided into normoalbuminuria, microalbuminuria, and macroalbuminuria subgroups based on urine albumin to creatinine ratio (ACR).* Results*. T2DM patients showed significantly lower serum adropin concentrations than those in the controls. T2DM patients with macroalbuminuria had significantly decreased serum adropin concentrations compared with the other three groups. In addition, T2DM patients with microalbuminuria showed lower serum adropin concentrations than those in patients with normoalbuminuria. Logistic regression analysis showed that serum adropin was correlated with decreased risk of developing T2DM and DN. Pearson correlation analysis indicated that serum adropin was negatively correlated with body mass index (BMI), blood urea nitrogen, creatinine, and ACR and positively correlated with glomerular filtration rate. Furthermore, multiple linear regression analysis showed that BMI and ACR were negatively correlated with serum adropin levels.* Conclusion*. Serum adropin concentrations are negatively associated with renal function. Adropin may be implicated in the pathogenesis of DN development.

## 1. Introduction

Diabetic nephropathy (DN), which is the leading cause of end-stage kidney disease, occurs in about 20–40% of diabetic patients [1]. Traditionally, metabolic and hemodynamic alterations caused by hyperglycemia and hypertension could contribute to renal injury in diabetic patients [2]. No effective treatments have been developed for DN. Currently available treatment options can only delay DN progression or turn to renal replacement therapies. Therefore, it is urgent to identify novel biomarkers for early diagnosis and develop some effective treatment strategies for patients at high risk.

Adropin is a newly identified metabolic hormone expressed in the liver and brain of mice [3]. Adropin is involved in the mechanism of increased adiposity, insulin resistance, and glucose and lipid metabolism [3]. Treatment with adropin can decrease blood glucose and improve insulin resistance in streptozotocin-induced type 2 diabetic rats [4]. It is reported that serum adropin levels decreased in patients with type 2 diabetes mellitus (T2DM) [5]. These findings indicate that adropin may be involved in the mechanism of T2DM. Adropin expression levels also increased in the kidney tissues of rats with streptozotocin-induced experimental diabetes [6]. Therefore, adropin may play a role in the pathogenesis of DN.

This study aims to determine the association of serum adropin with the development and progression of DN.

## 2. Materials and Methods

### 2.1. Patients

A total of 245 patients with T2DM were enrolled in this study. The patients were all diagnosed with T2DM in accordance with the American Diabetic Association criteria. T2DM patients were divided into three groups based on urine albumin to creatinine ratio (ACR): normoalbuminuria group (ACR < 30 mg/g; *n* = 110), microalbuminuria group (30 ≤ ACR ≤ 300 mg/g; *n* = 95), and macroalbuminuria group (ACR > 300 mg/g; *n* = 40). Subjects were excluded if they had severe cardiovascular diseases, malignant tumor, acute infection, or endocrine diseases. All patients received antidiabetic drug to control glycemia and were responsive to their respective therapies. The control group consisted of 81 healthy subjects who visited the general health check-up center of our hospital. None received medication or dietary supplements. And all the control subjects had no history of diabetes.

This study was approved by the hospital ethics board and all patients provided written informed consent.

### 2.2. Measurements

Serum was obtained from blood samples by centrifugation and stored at −80°C until analysis. Serum adropin concentrations were measured using an enzyme-linked immunosorbent assay kit (Phoenix Pharmaceuticals, Inc., USA) (coefficients of variations (CVs) for intra-assay: 5–7%; CVs for interassay: 12–15%; sensitivity: 0.3 ng/mL, detection limit range: 0.01–100 ng/mL). Metabolic syndrome (MetS) was defined using the criteria established in the third report of the National Cholesterol Education Program Expert Panel on Detection, Evaluation, and Treatment in Asian population (Adult Treatment Panel III) [7].

### 2.3. Statistical Analysis

Sample size was determined through power analysis using preliminary data obtained in our laboratory with the following assumptions: *α* of 0.05 (two-tailed) and power of 90%. A minimum of 11 subjects in the three T2DM subgroup and control group allowed the detection of difference in serum adropin concentrations. Data were expressed as means ± standard deviation or median (interquartile range). The differences of characteristics between three groups of T2DM patients and control subjects were compared using Chi-square tests, one-way ANOVA, or Kruskal-Wallis test. Logistic regression analysis was used to determine the risk factors for developing T2DM and DN. The correlations between serum adropin and other parameters were analyzed by Pearson correlation analysis. Multiple linear regression analysis was used to determine the contribution of various factors to serum adropin. *P* values less than 0.05 were considered statistically significant.

## 3. Results

### 3.1. Baseline Clinical Characteristics of T2DM Patients and Controls

The clinical parameters of T2DM patients and healthy controls are displayed in Table 1. T2DM patients showed elevated levels of systolic blood pressure (SBP), HbA1c, and the prevalence of MetS, as well as decreased levels of high-density lipoprotein cholesterol (HDL-C) compared with control subjects. In addition, significantly higher levels of blood urea nitrogen (BUN) and creatinine (Cr) and lower levels of glomerular filtration rate (GFR) were found in T2DM patients with macroalbuminuria compared with the other three groups.

### 3.2. Serum Adropin Concentrations

As shown in Table 1, serum adropin concentrations were significantly elevated in the control group compared with T2DM patients. Serum adropin concentrations were significantly reduced in T2DM patients with macroalbuminuria compared with those with normoalbuminuria and microalbuminuria. In addition, T2DM patients with microalbuminuria showed lower serum adropin concentrations than those with normoalbuminuria.

### 3.3. The Association of Serum Adropin Concentrations with T2DM

T2DM patients showed decreased serum adropin concentrations compared with healthy controls (2.88 (2.43–3.41) ng/mL versus 3.71 (2.82–4.56) ng/mL, *P* < 0.001). As presented in Table 2, logistic regression analysis showed that serum adropin was negatively correlated with T2DM (OR 0.282, 95% CI 0.195 to 0.406; *P* < 0.001). After adjusting for age and gender, serum adropin was associated with a decreased risk of developing T2DM (OR 0.274, 95% CI 0.190 to 0.396; *P* < 0.001). Simple logistic regression analysis showed that SBP, diastolic blood pressure (DBP), HDL-C, low-density lipoprotein cholesterol (LDL-C), BUN, the prevalence of MetS, and serum adropin showed a trend (*P* < 0.05) toward an association with T2DM. All of these variables were then entered into a backward stepwise multivariate logistic regression model. Multivariate logistic regression revealed that serum adropin remained a significant predictor of T2DM (OR 0.278, 95% CI 0.160 to 0.485; *P* < 0.001).

### 3.4. The Association of Serum Adropin Concentrations with DN

T2DM patients with microalbuminuria and macroalbuminuria were considered to have DN. Lower serum adropin concentrations were found in T2DM patients with DN compared with those without DN (2.73 (2.24–3.10) ng/mL versus 3.17 (2.63–3.68) ng/mL, *P* < 0.001). As shown in Table 3, logistic regression analysis showed that serum adropin was inversely associated with DN development (OR 0.288, 95% CI 0.183 to 0.453; *P* < 0.001). After adjusting for age and gender, serum adropin was still negatively correlated with the risk of developing DN (OR 0.285, 95% CI 0.180 to 0.452; *P* < 0.001). Simple logistic regression analysis showed that SBP, DBP, total cholesterol (TC), LDL-C, BUN, Cr, GFR, and serum adropin showed a trend (*P* < 0.05) toward an association with T2DM. All of these variables were then entered into a backward stepwise multivariate logistic regression model. Serum adropin remained a significant predictor of DN after the multivariate logistic regression (OR 0.270, 95% CI 0.160 to 0.455; *P* < 0.001).

### 3.5. The Association of Serum Adropin Concentrations with Other Clinical Characteristics

As presented in Table 4, Pearson correlation analysis showed that serum adropin was correlated with body mass index (BMI) (*r* = −0.215, *P* = 0.001), BUN (*r* = −0.245, *P* = 0.001), Cr (*r* = −0.285, *P* < 0.001), ACR (*r* = −0.358, *P* < 0.001), and GFR (*r* = 0.212, *P* < 0.001). Then a significant correlation of serum adropin with BMI (*r* = −0.210, *P* = 0.001), BUN (*r* = −0.219, *P* = 0.001), Cr (*r* = −0.256, *P* < 0.001), ACR (*r* = −0.352, *P* < 0.001), and GFR (*r* = 0.173, *P* = 0.007) was observed after adjusting for age and gender. Then we performed a multiple linear regression analysis. It showed that BMI (*β* = −0.198, *P* = 0.001) and ACR (*β* = −0.300, *P* < 0.001) were still negatively correlated with the serum adropin.

## 4. Discussion

Adropin is a newly discovered peptide correlated with energy regulation and obesity. Adropin also plays an important role in glucose metabolism and diabetes. Treatment with adropin could reduce blood glucose levels and insulin resistance and improve insulin sensitivity in a rat model of T2DM [4]. Adropin treatment enhanced glucose tolerance, ameliorated insulin resistance, and promoted the preferential use of carbohydrate over fat in diet-induced obese mice [8]. Adropin knockout mice showed increased adiposity and insulin resistance as well as dyslipidemia [9]. Maternal and fetal adropin levels in gestational diabetes mellitus (GDM) group were significantly lower than those in the control women [10]. Furthermore, patients with GDM showed significantly decreased serum adropin levels compared with the healthy controls [11]. T2DM patients exhibited relatively lower adropin levels than those of nondiabetic patients [5]. The present study demonstrated that serum adropin was correlated with decreased risk of developing T2DM after the logistic regression analysis. Hence, serum adropin should be utilized as a biomarker for assessing the risk of developing T2DM. However, no investigation has focused on the association of serum adropin with type 1 diabetes. Furthermore, we did not find a correlation of serum adropin with HbA1c which is a parameter for average blood glucose. Previous studies showed the role of adropin in glucose metabolism. Therefore, the association of serum adropin with blood glucose should be illustrated by future studies.

This study showed that decreased serum adropin concentrations were correlated with the development and progression of DN. Previous studies determined the role of adropin in DN. Adropin expression was detected in the kidney tissue of rats, including the glomerulus, peritubular interstitial cells, and peritubular capillary endothelial cells [6]. Adropin immunoreaction was enhanced in the kidney of diabetes-induced rats compared with that in the kidney of the controls [6]. The intensities of adropin immunoreactivity increased with diabetic severity [12]. However, the reason why serum adropin concentrations were low while adropin immunoreaction was high in diabetes or DN condition remains unknown. Further studies are required to explain this phenomenon.

The present study showed that serum adropin was correlated with renal function parameters such as BUN, Cr, and GFR. Adropin may be involved in the pathogenesis of kidney disease, not only DN. Future studies performed in patients with other kidney disease are needed to explain the precise role of adropin in kidney disease.

The precise role of adropin in DN mechanism remains unclear. Inflammation plays an important role in the development of DN. Adropin significantly decreased the mRNA expression levels of tumor necrosis factor-alpha (TNF-*α*) and interleukin 6 (IL-6) in the pancreas tissue of diabetic rats [4]. Circulating adropin level was negatively correlated with TNF-*α* level in women with polycystic ovarian syndrome [13]. Therefore, we hypothesize that adropin may play a protective role in DN development through anti-inflammatory effects.

We also evaluate the correlation of adropin with MetS. We found that serum adropin was correlated with BMI which is a parameter for obesity. However, no significant correlation of serum adropin with dyslipidemia, blood glucose, or blood pressure was found in the present study. Moreover, we defined MetS in the control and case groups and found that there is no correlation of serum adropin with MetS. Hence, adropin may be closely correlated with obesity. However, there is not enough evidence to conclude a correlation of adropin with MetS.

This study presents several limitations. First, the sample size was not sufficiently large to achieve definitive conclusions. Further studies with large populations are thus warranted. Second, our study utilized a cross-sectional design. Hence, the causative relation must be confirmed by future longitudinal studies.

In conclusion, serum adropin concentrations are negatively associated with renal function. Adropin may be implicated in the pathogenesis of DN development.

## Figures and Tables

**Table 1:** Clinical characteristics of T2DM patients and controls.

	Control	T2DM patients			
		Normoalbuminuria	Microalbuminuria	Macroalbuminuria	*P*
*N*	81	110	95	40	
Age (years)	58.35 ± 7.80	57.52 ± 11.33	58.45 ± 13.16	59.92 ± 10.78	0.701
Gender (M/F)	46/35	58/521	49/46	21/19	0.911
BMI (Kg/m^2^)	25.73 ± 2.84	26.06 ± 3.99	26.50 ± 3.52	26.33 ± 3.07	0.512
SBP (mmHg)	122.52 ± 11.28	135.50 ± 22.26^a^	147.63 ± 29.95^ab^	156.65 ± 23.26^abc^	<0.001
DBP (mmHg)	79.10 ± 7.45	80.45 ± 14.26	88.00 ± 19.60^ab^	86.08 ± 12.74^ab^	0.001
HbA1c (%)	4.76 ± 0.32	7.85 ± 1.43^a^	8.06 ± 1.23^a^	7.96 ± 1.67^a^	<0.001
TG (mmol/L)	2.08 ± 1.58	1.89 ± 1.10	2.16 ± 1.92	2.05 ± 1.11	0.626
TC (mmol/L)	5.23 ± 0.90	5.09 ± 1.07	5.45 ± 1.26^b^	5.32 ± 1.02	0.115
HDL-C (mmol/L)	1.49 ± 0.25	1.12 ± 0.24^a^	1.13 ± 0.21^a^	1.17 ± 0.33^a^	<0.001
LDL-C (mmol/L)	3.25 ± 0.54	3.39 ± 0.89	3.69 ± 1.03^a^	3.52 ± 0.80	0.006
BUN (nmol/L)	5.37 ± 1.18	5.33 ± 1.58	5.91 ± 1.98^b^	8.63 ± 3.94^abc^	<0.001
Cr (*μ*mol/L)	66.46 ± 10.37	65.27 ± 18.16	66.33 ± 21.27	115.47 ± 70.72^abc^	<0.001
ACR (mg/g)	—	15.94 ± 4.45	93.51 ± 80.98^b^	>300^bc^	<0.001
GFR (mL/min/1.73 m^2^)	101.77 ± 11.85	109.90 ± 38.96	109.13 ± 33.39	70.50 ± 35.65^abc^	<0.001
Adropin (ng/mL)	3.71 (2.82–4.56)	3.17 (2.63–3.68)^a^	2.85 (2.32–3.27)^ab^	2.56 (2.07–2.85)^abc^	<0.001
MetS (%)	4 (4.94%)	73 (66.36%)^a^	68 (71.56%)^a^	33 (82.5%)^a^	<0.001

^a^Significant versus control subjects.

^b^Significant versus T2DM patients with normoalbuminuria.

^c^Significant versus T2DM patients with microalbuminuria.

T2DM, type 2 diabetes mellitus; BMI, body mass index; SBP, systolic blood pressure; DBP, diastolic blood pressure; TG, triglycerides; TC, total cholesterol; HDL-C, high-density lipoprotein cholesterol; LDL-C, low-density lipoprotein cholesterol; BUN, blood urea nitrogen; Cr, creatinine; ACR, urine albumin to creatinine ratio; GFR, glomerular filtration rate; MetS, metabolic syndrome.

**Table 2:** Logistic regression analysis for determining the risk factor of developing T2DM.

Characteristics	Adjusting for age and gender		Simple logistic regression		Multiple logistic regression	
	OR (95% CI)	*P*	OR (95% CI)	*P*	OR (95% CI)	*P*
Age (years)	0.991 (0.965–1.017)	0.479	0.999 (0.977–1.022)	0.959	—	—
Gender (M/F)	1.498 (0.844–2.658)	0.167	1.201 (0.724–1.993)	0.477	—	—
BMI (kg/m^2^)	—	—	1.047 (0.973–1.128)	0.220	—	—
SBP (mmHg)	—	—	1.054 (1.036–1.072)	<0.001	1.087 (1.040–1.135)	<0.001
DBP (mmHg)	—	—	1.029 (1.008–1.051)	0.007	0.927 (0.870–0.988)	0.020
TG (mmol/L)	—	—	0.977 (0.830–1.150)	0.780	—	—
TC (mmol/L)	—	—	1.028 (0.815–1.297)	0.816	—	—
HDL-C (mmol/L)	—	—	0.004 (0.001–0.016)	<0.001	0.004 (0.001–0.030)	<0.001
LDL-C (mmol/L)	—	—	1.513 (1.091–2.099)	0.013	3.906 (1.950–7.823)	<0.001
BUN (nmol/L)	—	—	1.201 (1.037–1.391)	0.015	1.165 (0.888–1.528)	0.270
Cr (*μ*mol/L)	—	—	1.009 (0.998–1.021)	0.096	—	—
GFR (mL/min/1.73 m^2^)	—	—	1.001 (0.994–1.009)	0.750	—	—
Adropin (ng/mL)	0.274 (0.190–0.396)	<0.001	0.282 (0.195–0.406)	<0.001	0.246 (0.130–0.465)	<0.001
MetS	—	—	47.176 (16.64–133.78)	<0.001	12.422 (3.453–44.68)	<0.001

**Table 3:** Logistic regression analysis for determining the risk factor of developing DN.

Characteristics	Adjusting for age and gender		Simple logistic regression		Multiple logistic regression	
	OR (95% CI)	*P*	OR (95% CI)	*P*	OR (95% CI)	*P*
Age (years)	1.001 (0.978–1.024)	0.961	1.010 (0.989–1.031)	0.373	—	—
Gender (M/F)	1.167 (0.673–2.024)	0.583	1.036 (0.626–1.715)	0.891	—	—
BMI (kg/m^2^)	—	—	1.029 (0.960–1.103)	0.414	—	—
SBP (mmHg)	—	—	1.024 (1.012–1.035)	<0.001	1.024 (1.006–1.041)	0.008
DBP (mmHg)	—	—	1.030 (1.011–1.048)	0.001	1.007 (0.980–1.035)	0.618
HbA1c (%)	—	—	1.093 (0.911–1.311)	0.340	—	—
TG (mmol/L)	—	—	1.126 (0.931–1.362)	0.221	—	—
TC (mmol/L)	—	—	1.298 (1.027–1.641)	0.029	1.888 (0.710–5.020)	0.203
HDL-C (mmol/L)	—	—	1.419 (0.494–4.071)	0.515	—	—
LDL-C (mmol/L)	—	—	1.339 (1.010–1.775)	0.043	0.665 (0.203–2.176)	0.500
BUN (nmol/L)	—	—	1.346 (1.165–1.555)	<0.001	1.380 (1.111–1.714)	0.004
Cr (*μ*mol/L)	—	—	1.016 (1.005–1.028)	0.004	1.003 (0.982–1.024)	0.802
GFR (mL/min/1.73 m^2^)	—	—	0.991 (0.984–0.999)	0.019	1.010 (0.998–1.021)	0.095
Adropin (ng/mL)	0.285 (0.180–0.452)	<0.001	0.288 (0.183–0.453)	<0.001	0.270 (0.160–0.455)	<0.001
MetS	—	—	1.506 (0.865–2.621)	0.148	—	—

**Table 4:** The correlation between serum adropin concentrations and various parameters.

Parameters	Pearson correlation analysis		Adjusting for age and gender		Multiple regression analysis	
	*r*	*P*	*r*	*P*	*β*	*P*
Age (years)	−0.129	0.044				
Gender (M/F)	0.060	0.352				
BMI (Kg/m^2^)	−0.215	0.001	−0.210	0.001	−0.198	0.001
SBP (mmHg)	−0.069	0.284				
DBP (mmHg)	−0.067	0.296				
HbA1c (%)	−0.018	0.781				
TG (mmol/L)	0.072	0.260				
TC (mmol/L)	0.049	0.444				
HDL-C (mmol/L)	−0.026	0.689				
LDL-C (mmol/L)	0.028	0.668				
BUN (nmol/L)	−0.245	<0.001	−0.219	0.001	−0.029	0.766
Cr (*μ*mol/L)	−0.285	<0.001	−0.256	<0.001	−0.105	0.328
ACR (mg/g)	−0.358	<0.001	−0.352	<0.001	−0.300	<0.001
GFR (mL/min/1.73 m^2^)	0.212	<0.001	0.173	0.007	0.006	0.941
MetS	0.006	0.921	—	—	—	—
